# Tenofovir as Rescue Therapy Following Clinical Failure to Lamivudine in Severe Acute Hepatitis B

**DOI:** 10.4084/MJHID.2013.035

**Published:** 2013-06-03

**Authors:** Jurgen Gerada, Elaine Borg, Denise Formosa, Rosalie Magro, James Pocock

**Affiliations:** Division of Gastroenterology, Mater Dei Hospital, Malta

## Abstract

Acute hepatitis B (AHB) is a self-limiting condition in more than 95% of cases. Treatment is however recommended in patients with severe AHB (<1% of cases), aiming to prevent liver failure and death. Various nucleos(t)ide analogues (NA) have been found to be effective in severe AHB, although NA-resistant strains causing AHB have been also recently reported. The use of tenofovir in severe AHB has only been described in 3 cases (1 adult and 1 infant with HBV mono-infection, 1 adult with HBV/HIV co-infection). We hereby report a 47-year-old treatment-naïve male, who developed severe AHB and was initially treated with lamivudine (LMV). Initial rapid biochemical response was followed by biochemical breakthrough after 9 days, suggesting LMV resistance. Rescue therapy with ‘add-on’ tenofovir brought about a sustained improvement in biochemical, serological and virological markers until HBsAg was lost after 4 months. Thus, this is the second adult HBV mono-infected patient, who responded successfully to tenofovir in severe AHB.

## Introduction

Acute hepatitis B (AHB) is a self-limiting condition in more than 95% of cases; however, in less than 1% of cases, it may progress to liver failure necessitating liver transplantation. Treatment for AHB is usually not required, however international bodies recommend to commence treatment in patients with fulminant or severe acute hepatitis B^1^,^2^ - the latter defined by any two of the following three criteria: serum bilirubin equal to or more than 10mg/dL, INR equal to or more than 1.6 and hepatic encephalopathy^3^ - aiming at preventing progression to liver failure. As Interferon therapy is contraindicated in this scenario due to the risk of worsening hepatitis and frequent side effects,^2^ both AASLD and EASL have recommended the use of a nucleos(t)ide analogue (NA) in severe AHB.^2^ To date, however, there is still no evidence which NA is best in this setting, as randomized controlled trials comparing the efficacy of different NAs are lacking, owing to the difficulty in enrolling such non-frequent patients in two arms and being adequately numerous.

Despite lamivudine (LMV) being the most widely used antiviral in severe AHB with encouraging results,^4^ we have seen recently the emergence of LMV resistant strains in treatment-naïve patients causing primary drug resistance and thus requiring newer antivirals such as entecavir for rescue therapy.^5^ Documented use of these newer antivirals, especially tenofovir, in severe AHB, as first line or rescue therapy has been as yet very limited. To date, there have been only 3 published cases of fulminant or severe AHB treated with tenofovir.^6^–^8^ These include two adult patients (one with HBV mono-infection^6^ and one with HBV/HIV co-infection^7^) and one 4-month old HBV mono-infected infant.^8^ The two mono-infected patients cleared the virus with tenofovir and survived, while the HBV/HIV co-infected patient succumbed to the disease. We hereby report the second case in the English literature, where tenofovir was successfully used in an adult non-HIV patient with severe AHB, who developed clinical LMV failure after few days of LMV monotherapy.

## Case Report

A 47-year-old male, known to suffer from asthma, presented to the accident and emergency department with a nine day history of jaundice, epigastric pain, nausea, lethargy and decreased appetite. This was accompanied by a three day history of low grade fever, dark urine and pale stools. His alcohol intake ranged between 8 –10 units per week and he did not report taking any new medications or any recent traveling. Although revealing no illicit intravenous drug use, he frequently smoked marijuana recreationally, as well as a packet of cigarettes daily. He was in a stable ten-year relationship with a male partner and both of them had checked their viral status one month earlier, all of which, including hepatitis B, C and HIV, were negative. His past medical history was unremarkable.

On examination, the patient had a Glasgow coma scale of 15 and was oriented to time, place and person. He was jaundiced, had a temperature of 37.5°C and was haemodynamically stable. There were no stigmata of chronic liver disease and a hepatic flap could not be elicited. He was tender in the right upper quadrant and had a palpable hepatomegaly four centimetres below the right costal margin. No associated splenomegaly or ascites were present. His cardiorespiratory system was normal.

Laboratory investigations showed a white cell count 7.6 × 10^9^/L, haemoglobin 15.3g/dL, platelet count 100 × 10^9^/L, normal renal function (estimated glomerular filtration rate (eGFR) 81mls/min/1.72m^2^), normal glucose and amylase. Liver function tests revealed a hepatitic picture with a total bilirubin 10.8mg/dL (direct bilirubin 10.5mg/dL), alkaline phosphatase (ALP) 367 U/l, alanine aminotransferase (ALT) 3258 U/l, gamma glutamyl transferase (GGT) 1072 U/l and an abnormal synthetic function with an International normalized ratio (INR) of 1.63 and albumin 33.3g/L. Serologic tests were positive for hepatitis B surface antigen (HBsAg), IgM antibodies to hepatitis B core antigen (anti-HBc) and hepatitis B e antigen (HBeAg). Antibodies to hepatitis B e antigen (anti-HBe) were absent. HBV DNA was detected in the serum using polymerase chain reaction (PCR). Tests for IgM antibodies to hepatitis A virus, hepatitis C virus, human immunodeficiency virus and cytomegalovirus were all negative. The source of this acute HBV infection could not be confirmed. An ultrasound of the abdomen showed two liver haemangiomas, one measuring 1.0 × 1.3cm in the left lobe and the other measuring 1.4 × 1.4cm in the right lobe. No gallbladder stones were present and the biliary tree was of normal caliber.

**Figure 1:**
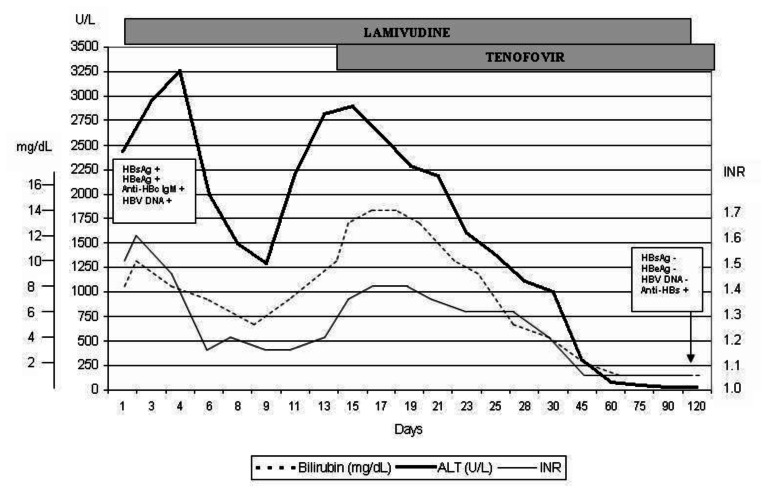
Biochemical and viral serological markers during the course of the disease, before and after the introduction of tenofovir. ALT, Alanine aminotransferase (U/L); INR, International normalized ratio; HBsAg, hepatitis B surface antigen; HBeAg, Hepatitis B e antigen; Anti-HBc IgM, anti-hepatitis B core IgM antibody; HBV DNA, Hepatitis B DNA; Anti-HBs, anti-hepatitis B surface antibody.

Initially, he was started on N-acetyl cysteine at a dose of 100mg/kg in 1L 5% dextrose every 16 hours. On day 2, after repeating and confirming the total and direct bilirubin to be ≥10mg/dL and INR ≥1.6, in accordance with having fulfilled two out of the three criteria for the diagnosis of severe AHB (clinical encephalopathy was not present), antiviral therapy with lamivudine 100mg daily was then added. A dramatic improvement in liver enzymes and INR was observed, however this was only sustained for the first 9 days (total bilirubin decreased to 5.5mg/dL, ALT 1237U/l and INR 1.15) (Figure 1). On day 10, deterioration in the biochemical parameters was once again noted, and on day 14, since the deterioration persisted (total bilirubin increased to 13.9mg/dL, ALT 2896U/l and INR 1.38), tenofovir 245mg daily was commenced. This brought about a gradual improvement in liver enzymes and synthetic function. Total normalization of the liver biochemical parameters was achieved after 3 months (Figure 1). At this point, lamivudine was decreased to 100mg every alternate day. After a further one month, the patient had managed to achieve anti-HBs seroconversion. At this point, lamivudine was therefore stopped and tenofovir was eventually tailed down over another two months. Renal function and serum phosphate levels remained intact throughout this period (eGFR 87mls/min/1.72m^2^, no proteinuria on urinalysis and serum phosphate 1.22mmol/l). PCR for HBV DNA was undetectable at this stage and anti-HBsAg antibody titres were positive at 212mIU/ml.

## Discussion

The transplant-free survival rates in fulminant or severe AHB with or without antiviral therapy have been studied by various groups.^4^,^9^,^10^ In a pooled analysis by Tillmann et al., the transplant-free survival rate in untreated severe AHB was 45.7%, whilst that in LMV-treated severe AHB patients was 78.2%.^11^ The rationale of treating patients with fulminant or severe AHB is thus justified, with NAs being the preferred option,^1^,^2^ aiming at reducing mortality. Moreover, in patients requiring liver transplantation, treating severe AHB offers a higher chance of HBV DNA suppression, thereby reducing the risk of reinfection in the graft.^11^ Our patient fulfilled two out of the three diagnostic criteria of severe AHB (bilirubin 10.8mg/dL, INR 1.63) and was thus deemed a candidate for treatment, particularly in our centre where liver transplantation facilities are lacking.

LMV has been the most widely studied antiviral in severe AHB. It has been shown to be safe and effective,^4^ with a rapid onset of action^11^ and able to decrease HBV DNA load, preventing progression to liver failure^12^ and reducing mortality.^11^ This has been clearly demonstrated when LMV was started earlier on, prior to the development of overt encephalopathy, highlighting the importance of significant prolonged prothrombin time as being a crucial marker of impaired liver function.^11^ However, too-early administration of lamivudine has also been found to reduce the rates of HBsAg and HBeAg seroconversion.^13^ The low genetic barrier and development of resistance of LMV is perhaps its major shortcoming, which has thoroughly been described in chronic hepatitis B, especially when given as monotherapy.^14^ On the contrary, in AHB, where the treatment duration is anticipated to be short, LMV resistance seldom occurs, especially during the first 36 weeks of therapy, as described in a large series by Atkins et al..^15^ In fact, AASLD recommend LMV or telbivudine monotherapy as first line therapy in severe AHB infection when the anticipated duration of treatment is short.^2^

Recently, however, LMV-resistant strains in AHB have started to emerge as well, even in treatment-naïve patients, necessitating the need to switch therapy to a newer NA with a higher barrier to resistance. With such resistant strains becoming more prevalent, the importance of identifying the genotype and its resistance pattern prior to commencing therapy has become more pertinent and is therefore encouraged. The first two published cases of LMV-resistant strains in severe AHB by a German group in 2008, reported primary LMV resistance and were salvaged successfully by switching to entacavir or to ‘add-on’ adefovir respectively.^5^ Since then, both Japan and China have reported cases of LMV-resistant strains in AHB.^16^–^18^ In the Japanese study, the incidence of these strains was 4.44% (2/45).^16^ The two cases reported, one with subgenotype C2 and the other with subgenotype A2, developed a self-limiting hepatitis and a severe AHB respectively, who had a favorable outcome with LMV and steroids.^16^ The incidence of LMV-resistant strains in the Chinese series was however reported as 7% (14/201), all of whom were NA-untreated patients with AHB, with the predominant genotype being genotype C (10/14).^17^ They reported eleven cases of LMV-resistant strains, with the most frequent mutations being rtM204I and/or rtM204V, two cases of adefovir-resistant strains and one case of entacavir-resistant strain.^17^ In a further Chinese study published last year, where the incidence of LMV-resistant strains was reported as 4.7% (11/234) (nine with self-limiting hepatitis and two with severe AHB), genotype C was once again the predominant genotype (8/11).^18^ This Chinese study was the first, however, to demonstrate that such LMV-resistant strains may progress into a chronic infection following an acute episode.^18^ This occurred in one patient with LMV-resistant AHB genotype C, who developed chronic occult HBV infection.^18^ Identification of more mutant strains to adefovir, lamivudine and entecavir were reported this year by Baxa et al. in three symptomatic AHB patients,^19^ while Morando et al. detected LMV and telbivudine relevant mutations in a patient with severe AHB genotype C.^6^ To date, as yet, there are no reported tenofovir-resistant viral mutants causing AHB.

The use of NAs other than LMV in AHB, such as adefovir, entecavir or tenofovir as first line monotherapy, rescue ‘add on’ therapy or combined therapy with LMV, is very limited. The few reported cases to date that have used adefovir or entacavir include primary monotherapy use of entacavir in a case series of six German patients^20^ and two Italian patients,^21^,^22^ and rescue therapy with adefovir and entacavir in the two German cases mentioned above.^5^ All these cases had favorable outcomes. Tenofovir use in severe AHB has been only reported in three cases; two adult patients (one with HBV mono-infection^6^ and another with HBV/HIV co-infection^7^) and one HBV mono-infected 4-month old infant.^8^ The patient with co-infection succumbed to the disease despite therapy but the other two cases achieved full viral suppression and seroconversion and survived without requiring transplantation. Baxa et al. reported the use of tenofovir in four American patients with symptomatic, but not severe, AHB, with resolution of the infection occurring in just two.^19^ The outcome of the other two were unfortunately not reported.^19^ Although tenofovir is a highly effective antiviral with a high barrier to resistance, caution needs to be exercised in its use due to the risk, albeit low, of nephrotoxicity and bone toxicity. Madeddu et al. reported a 2.5% prevalence of nephrotoxicity in HIV patients receiving combination antiretroviral therapy including tenofovir,^23^ while De Socio et al. reported a case of tenofovir-induced hypophosphatemic osteomalacia, which resolved on discontinuation of the drug.^24^

Our case is the second documented case where tenofovir was successfully used in a non-HIV adult treatment-naïve patient with severe AHB. Initial rapid biochemical improvement suggested satisfactory LMV response; however the biochemical breakthrough that occurred on day 9 of treatment suggested the development of LMV resistance. Lack of genomic sequence analysis facilities in our institution precluded us from identifying the genotype of this strain and the mutation that occurred in the HBV polymerase gene. It is difficult therefore to establish whether there were pre-existing mutant HBV strains, which emerged under LMV pressure or whether this was a *de novo* generation of a mutant quasispecies after LMV exposure. It thus seemed reasonable at this stage to use one of the most potent NAs with the highest barrier to resistance as a rescue therapy, as lack of identification of the underlying genetic mutation/s precluded us from understanding whether this was a LMV-only resistant virus or a multi-drug resistant virus. Our choice to use tenofovir, instead of entecavir, was based on the fact that both the efficacy and the genetic barrier to entecavir are significantly reduced in the presence of LMV-resistant mutations.^25^,^26^ Moreover, entecavir is associated with increased drug resistance in LMV-refractory chronic hepatitis B patients.^27^ On the contrary, tenofovir has been shown to be highly effective against LMV-resistant HBV virus,^28^,^29^ and as yet there are no reports of tenofovir resistance, and thus this seemed to be the best option in our patient. This proved to be successful by suppressing HBV DNA and bringing about HBsAg loss after four months with later seroconversion to anti-HBs, without the development of any renal complications. In keeping with the AASLD guidelines,^2^ where treatment can be stopped once HBsAg clearance has been achieved, our group tailed down tenofovir gradually over the following few weeks.

In conclusion, we have hereby reported the second case of a treatment-naïve adult non-HIV patient with severe AHB, who responded successfully to tenofovir following clinical failure to LMV monotherapy, suggesting LMV resistance. Tenofovir seems safe and effective in this setting and we thus concur with Morando et al. that tenofovir may be a suitable option for the treatment of severe AHB. The importance of identifying the viral genotype and its resistance pattern at baseline prior to commencing treatment in order to guide the appropriate choice of antiviral should be emphasized.
